# Network Analysis of Burnout and Safety Competence Among Oncology Nurses: A Secondary Study to Identify Bridge Targets for Precision Interventions

**DOI:** 10.1155/jonm/5604987

**Published:** 2026-03-23

**Authors:** Fengyan Ma, Zhao Luo, Yajing Zhu, Lu Liu, Helin Chen, Yan Liu, Fan Zhang

**Affiliations:** ^1^ Department of Thoracic Surgery, National Cancer Center/National Clinical Research Center for Cancer/Cancer Hospital, Chinese Academy of Medical Sciences and Peking Union Medical College, Beijing, China, cacms.ac.cn; ^2^ Department of Thoracic Surgery, Peking Union Medical College Hospital, CAMS & PUMC, Beijing, China, pumch.cn; ^3^ Nursing Department, National Cancer Center/National Clinical Research Center for Cancer/Cancer Hospital, Chinese Academy of Medical Sciences and Peking Union Medical College, Beijing, China, cacms.ac.cn

**Keywords:** bridge expected influence, burnout, latent profiles, network analysis, nurse safety behavior

## Abstract

**Background:**

Occupational burnout poses a persistent threat to nurses’ mental health and the quality of patient care. Emerging evidence indicates that burnout is not a uniform phenomenon but manifests in distinct psychological patterns. Yet, how these diverse burnout experiences interact with safety‐related factors is insufficiently understood. Network analysis offers a systems‐level perspective to uncover interconnections among psychological and safety variables and to pinpoint potential bridge nodes for targeted interventions.

**Aim:**

This study sought to map the network architecture linking psychological and safety‐related factors among nurses across different burnout profiles, to identify profile‐specific central and bridge nodes, and to examine their associations with safety behaviors.

**Methods:**

A total of 2092 nurses were included. This study was a secondary analysis based on a previously established dataset in which three distinct burnout profiles were identified using latent profile analysis: the High Achievement Stable Group (Class 1, 70.3%), the High Efficiency Contradictory Group (Class 2, 6.6%), and the High Pressure Adaptive Group (Class 3, 23.1%). Psychological–safety networks were estimated for both the overall sample and each subgroup using the EBICglasso model. Centrality and bridging indices were computed via expected influence and bridge expected influence, followed by network comparison tests to evaluate structural variations across profiles.

**Results:**

In the overall network, “skills” (B4) exhibited the greatest centrality, whereas “personal accomplishment” (A3) and “knowledge” (B1) consistently functioned as pivotal bridge nodes across profiles. Although bridge configurations differed slightly among classes, A3 and B1 remained the principal connectors integrating psychological and safety communities. Significant structural differences were detected between Classes 2 and 1 (M test, *p* < 0.001) and between Classes 3 and 1 (M test, *p* < 0.001; S test, *p* = 0.002), with pronounced discrepancies in the edge patterns surrounding A3 and B1.

**Conclusions:**

The burnout–safety networks revealed distinct structural configurations across nurse subgroups. Identifying profile‐specific bridge nodes offers practical guidance for precision interventions that enhance safety behaviors and foster occupational resilience.

## 1. Background

Nurse burnout has become a widespread and increasingly severe occupational health issue globally, with an estimated prevalence of approximately 30% and a continuing upward trend [1]. Burnout is a psychological syndrome resulting from prolonged work‐related stress, characterized primarily by emotional exhaustion, depersonalization, and reduced personal accomplishment [2, 3]. Within the clinical nursing field, oncology nurses are at particularly high risk due to the dual burden of high‐intensity clinical demands and sustained emotional exposure to patient suffering, death, and end‐of‐life care [4–6].

Burnout poses a substantial threat to patient safety. Previous studies have demonstrated robust associations between nurse burnout and compromised safety culture, increased adverse events (e.g., medication errors, patient falls, and hospital‐acquired infections), and reduced patient satisfaction [7–9]. Evidence further suggests that burnout dimensions exert differential effects on safety outcomes, with emotional exhaustion and depersonalization showing stronger negative associations than reduced personal accomplishment [7]. These findings imply that burnout may influence nursing safety through multiple, dimension‐specific pathways rather than a single uniform mechanism.

Importantly, burnout is a heterogeneous phenomenon. Our previous latent profile analysis (LPA) identified three distinct burnout profiles among oncology nurses, revealing substantial differences in the configuration of core burnout dimensions [10]. This heterogeneity suggests that nurses with different burnout profiles may rely on distinct mechanisms to maintain patient safety, underscoring the need to move beyond variable‐centered approaches.

Within the nursing safety framework, safety competence and safety behavior represent two closely related but conceptually distinct components. Safety competence refers to nurses’ integrated capacity to recognize risks, apply preventive strategies, and manage safety‐related problems, encompassing knowledge, system awareness, attitudes, and skills [11]. Safety behavior reflects the enactment of this competence in clinical practice, including adherence to protocols, proactive reporting, teamwork, and protective actions [12, 13]. Clarifying how these components interact under different burnout profiles is essential for developing targeted safety interventions.

This study integrates network analysis with LPA. Network analysis conceptualizes burnout dimensions, safety competence components, and safety behaviors as an interconnected system, enabling the identification of central components that exert disproportionate influence and bridge components that link psychological states with behavioral outcomes [14, 15]. By comparing network structures across distinct burnout profiles, this approach allows for the examination of profile‐specific safety mechanisms that cannot be captured by traditional linear models. Such insights may provide an empirical basis for stratified interventions aimed at simultaneously promoting nurse well‐being and patient safety.

### 1.1. Theoretical Framework and Research Hypotheses

Guided by Social Cognitive Theory (SCT), this study conceptualizes nurse burnout, safety competence, and safety behavior as dynamically interacting components within a reciprocal psychological–behavioral system [16]. Burnout represents an internal psychological state shaped by prolonged occupational stress, safety competence reflects nurses’ cognitive and motivational capacity under specific organizational and environmental conditions, and safety behavior constitutes the observable execution of safe practices in clinical settings. Emotional exhaustion may impair attention, vigilance, and work engagement, thereby undermining nurses’ ability to identify and respond to safety risks [17]. Depersonalization may disrupt communication and teamwork, weakening the interpersonal processes essential for safe care delivery [18]. Reduced personal accomplishment may diminish self‐efficacy and intrinsic motivation, lowering nurses’ willingness to engage in proactive safety behaviors [19]. Through these pathways, burnout may compromise safety competence directly and affect patient safety indirectly via altered safety behavior.

Given the multidimensional nature of safety competence, its components may function differently across burnout profiles. Knowledge and skills may serve as proximal determinants of safety behavior, whereas attitudes and system‐related factors may operate as connecting elements within organizational and team contexts [20, 21]. Accordingly, examining safety competence at the dimensional level is critical for understanding how burnout‐related psychological states translate into behavioral outcomes.

Integrating SCT with a network perspective enables the exploration of how these components are structurally interrelated across burnout profiles. By identifying profile‐specific core and connecting elements within the psychological–safety system, this study aims to describe the patterns linking burnout dimensions and safety‐related behaviors, providing evidence to inform the design of tailored, profile‐sensitive interventions.

#### 1.1.1. Study Hypotheses

 H1: Burnout, safety competence, and safety behavior among nurses are interconnected via multidimensional relationships, with different components showing varying centrality within the network.
 H2: Safety competence functions as a bridge between burnout and safety behavior, and its subcomponents differ in influence.
 H3: Network structure, core nodes, and bridge nodes differ across burnout profiles.

## 2. Methods

### 2.1. Participants, Settings, and Procedure

This study represents a secondary analysis of cross‐sectional data. The detailed recruitment and data collection procedures for this sample have been previously published in a study reporting its LPA [22]. Briefly, a convenience sample of 2092 nurses was recruited from April to June 2023 from tertiary cancer hospitals across 12 provinces in China. Eligible participants were registered clinical nurses aged 18 years or older. Nurses were excluded if they were currently on maternity or sick leave, or if they were visiting for advanced study or clinical internship.

After securing permissions from hospital management, the survey was administered online via “Questionnaire Star” (Wenjuanxing), with an estimated completion time of 10–15 min. Informed consent was obtained, and data quality was ensured via single IP address restrictions and mandatory responses. Of the 2240 questionnaires distributed, 2149 were returned. A total of 57 questionnaires were excluded based on predefined quality control criteria: those with more than 20% missing data and those completed in less than 10 min, as rapid completion likely indicated inattentive or rushed responses. After applying these criteria, the final valid sample included 2092 nurses, resulting in a valid response rate of 93.4%.

### 2.2. Variables and Instruments

General Information: Participants’ demographic and professional characteristics were collected, including gender, age, education level, job position, title, employment form, years of service, monthly income, marital status, weekly working hours, career‐related adverse events, and safety training.

#### 2.2.1. Burnout

We used the Maslach Burnout Inventory‐Human Services Survey (MBI‐HSS) [2], which has been validated in Chinese nurses [23]. It comprises 22 items across three dimensions: emotional exhaustion, depersonalization, and personal accomplishment, rated on a 7‐point Likert scale (0–6). In this study, Cronbach’s alpha was 0.934 for the overall scale and ranged from 0.865 to 0.934 across subscales.

#### 2.2.2. Patient Safety Competence

The Patient Safety Competency Self‐Rating Scale of Nurses (PSC‐SSN) [12] was used. This 29‐item scale assesses four dimensions: knowledge (10 items), system (8 items), attitude (5 items), and skills (6 items) on a 5‐point Likert scale (1–5). Cronbach’s alpha in this study was 0.970 overall, with subscales ranging from 0.812 to 0.934.

#### 2.2.3. Nurse Safety Behaviors

The Nurse Safety Behavior Questionnaire (NSBQ) [24] measured safety behaviors. This 12‐item, one‐dimensional scale uses a 5‐point Likert scale (1–5). In the current sample, Cronbach’s alpha was 0.910.

### 2.3. Data Analysis

All analyses were conducted using R (Version 4.4.3) and SPSS 24.0. Descriptive statistics were used to summarize demographic and psychological variables, with continuous data expressed as mean ± standard deviation (SD) and categorical data as frequencies and percentages. The latent profile classification adopted the three‐class solution identified and validated in our previous study: High Achievement Stable Group, High Efficiency Contradictory Group, and High Pressure Adaptive Group.

Based on this latent profile solution, the three burnout profiles were characterized as follows. Class 1 (High Achievement Stable Group, 70.3%) exhibited low emotional exhaustion and depersonalization, coupled with high personal accomplishment, and served as the low‐burnout reference group. Class 2 (High Efficiency Contradictory Group, 6.6%) showed elevated emotional exhaustion and depersonalization while maintaining high personal accomplishment, reflecting a “high‐demand–high‐efficacy” pattern. Class 3 (High Pressure Adaptive Group, 23.1%) displayed high emotional exhaustion and depersonalization accompanied by reduced personal accomplishment, indicating psychological resource depletion and a high‐stress adaptive state.

Psychological–safety networks were estimated for the total sample and each latent class using the EBICglasso model [25]. Network density and average edge weight were computed to quantify global connectivity, and node centrality was assessed using expected influence (EI) [26]. Bridge centrality indices were calculated using the networktools package. Nodes with bridge expected influence (BEI) values above the 80th percentile were identified as key connectors between distinct psychological and safety communities [27]. This approach enabled the identification of bridge nodes that play a pivotal role in transmitting influence across dimensions [28].

#### 2.3.1. Network Stability Assessment

To evaluate the accuracy and stability of the results, a bootstrapping procedure was performed using the “bootnet” package. The network model was re‐estimated 1000 times with nonparametric bootstrap samples, and 95% confidence intervals (95% CIs) of the edge weights were calculated to assess edge stability. Node stability was examined by iteratively removing portions of the sample during the bootstrap process, and stability coefficients were then computed. The node stability coefficient represents the correlation between network parameters in the resampled and original networks multiplied by the proportion of retained cases. When the coefficient exceeds 0.7, the node estimates are considered highly stable, and a minimum acceptable threshold is 0.25; values above 0.5 indicate relatively stable node indices [29].

Network differences among classes were examined using the Network Comparison Test (NCT), which was applied to evaluate both structural invariance (M) and global strength invariance (S) across groups.

### 2.4. Ethical Consideration

This study is a secondary analysis of an existing, anonymized dataset. The original data collection procedures were conducted in strict accordance with the Declaration of Helsinki and Chinese national ethical regulations. As detailed in the primary publication [22], the original study protocol was deemed exempt from prior ethical review by institutional guidelines (in accordance with the Measures for the Ethical Review of Life Sciences and Medical Research Involving Humans, 2023). All participants provided written informed consent during the original data collection, and all data used in this secondary analysis were fully anonymized.

## 3. Results

### 3.1. Sample Characteristics and Latent Profile Groups

This analysis was conducted on a dataset of 2092 participants, which was previously utilized in a study establishing their latent profiles. Based on that LPA, the sample was segmented into three distinct profiles: the “High Achievement Stable Group” (Class 1, 70.3%, *n* = 1475), the “High Efficiency Contradictory Group” (Class 2, 6.6%, *n* = 137), and the “High Pressure Adaptive Group” (Class 3, 23.1%, *n* = 480).

The demographic and professional characteristics of the total sample, along with comparisons across the three profiles, are presented in Table 1. Significant differences (*p* < 0.05) were observed between the groups for age, gender, job position, title, years of service, employment form, monthly income, weekly working hours, adverse incident experience, and safety training courses.

**TABLE 1:** Demographic information on survey respondents.

Variable	Number	High Achievement Stable Group	High Efficiency Contradictory Group	High Pressure Adaptive Group	Statistic	*p*
		*n* = 1475	*n* = 137	*n* = 480		
Age (years), mean ± SD	31.89 ± 7.73	32.06 ± 8.09	30.66 ± 7.70	31.75 ± 6.50	*F* = 3.63	**0.027**
Gender, *n* (%)					*χ* ^2^ = 31.97	**< 0.001**
Male	104 (4.97)	51 (3.46)	18 (13.14)	35 (7.29)		
Female	1988 (95.03)	1424 (96.54)	119 (86.86)	445 (92.71)		
Education level, *n* (%)					*χ* ^2^ = 9.47	0.149
Junior college	357 (17.07)	273 (18.51)	16 (11.68)	68 (14.17)		
Faulty‐to‐undergraduate	246 (11.76)	175 (11.86)	17 (12.41)	54 (11.25)		
Undergraduate	1461 (69.84)	1010 (68.47)	102 (74.45)	349 (72.71)		
≥ Postgraduate	28 (1.34)	17 (1.15)	2 (1.46)	9 (1.88)		
Job position, *n* (%)					*χ* ^2^ = 11.85	**0.018**
None	1821 (87.05)	1292 (87.59)	120 (87.59)	409 (85.21)		
Tutor	164 (7.84)	102 (6.92)	16 (11.68)	46 (9.58)		
Head nurse	107 (5.11)	81 (5.49)	1 (0.73)	25 (5.21)		
Title, *n* (%)					*χ* ^2^ = 34.18	**< 0.001**
Nurse	536 (25.62)	426 (28.88)	26 (18.98)	84 (17.50)		
Nurse practitioner	833 (39.82)	547 (37.08)	71 (51.82)	215 (44.79)		
Senior nurse or head nurse and above	723 (34.56)	502 (34.03)	40 (29.20)	181 (37.71)		
Years of service, *n* (%)					*χ* ^2^ = 55.88	**< 0.001**
< 4	503 (24.04)	388 (26.31)	31 (22.63)	84 (17.50)		
4 ∼	862 (41.20)	546 (37.02)	72 (52.55)	244 (50.83)		
10 ∼	500 (23.90)	350 (23.73)	31 (22.63)	119 (24.79)		
20 ∼	227 (10.85)	191 (12.95)	3 (2.19)	33 (6.88)		
Employment form, *n* (%)					*χ* ^2^ = 14.30	**0.006**
Formal incorporation	350 (16.73)	272 (18.44)	14 (10.22)	64 (13.33)		
Contract employment	1536 (73.42)	1050 (71.19)	111 (81.02)	375 (78.12)		
Personnel agency	206 (9.85)	153 (10.37)	12 (8.76)	41 (8.54)		
Monthly income (RMB), *n* (%)					*χ* ^2^ = 43.20	**< 0.001**
1000 ∼	320 (15.30)	214 (14.51)	30 (21.90)	76 (15.83)		
6000 ∼	1209 (57.79)	806 (54.64)	89 (64.96)	314 (65.42)		
10,000 ∼	563 (26.91)	455 (30.85)	18 (13.14)	90 (18.75)		
Marital status, *n* (%)					*χ* ^2^ = 1.52	0.467
Married	1263 (60.37)	882 (59.80)	80 (58.39)	301 (62.71)		
Single	829 (39.63)	593 (40.20)	57 (41.61)	179 (37.29)		
Weekly working hours, *n* (%)					*χ* ^2^ = 116.98	**< 0.001**
≤ 40	1604 (76.67)	1224 (82.98)	74 (54.01)	306 (63.75)		
> 40	488 (23.33)	251 (17.02)	63 (45.99)	174 (36.25)		
Adverse incident experience, *n* (%)					*χ* ^2^ = 87.95	**< 0.001**
None	1169 (55.88)	921 (62.44)	51 (37.23)	197 (41.04)		
Yes	923 (44.12)	554 (37.56)	86 (62.77)	283 (58.96)		
Safety training courses, *n* (%)					*χ* ^2^ = 17.36	**< 0.001**
Attended	1976 (94.46)	1412 (95.73)	122 (89.05)	442 (92.08)		
Not attended	116 (5.54)	63 (4.27)	15 (10.95)	38 (7.92)		

*Note:* F: ANOVA, *χ* ^2^: chi‐square test. Bold values indicate statistical significance (*p* < 0.05).

Abbreviation: SD, standard deviation.

Specifically, the “High Efficiency Contradictory Group” (Class 2) had a significantly higher proportion of males (13.14%) compared to the “High Achievement Stable Group” (Class 1, 3.46%). This group (Class 2) also had a notably higher rate of contract employment (81.02%), the lowest proportion of nurses with 20 or more years of service (2.19%), and the lowest proportion earning over 10,000 RMB monthly (13.14%).

Furthermore, both the “High Efficiency Contradictory Group” (Class 2) and the “High Pressure Adaptive Group” (Class 3) reported significantly more demanding work conditions than Class 1. They had a much higher prevalence of working > 40 h per week (45.99% and 36.25%, respectively, vs. 17.02% in Class 1) and a significantly higher likelihood of having experienced an adverse incident in their career (62.77% and 58.96%, respectively, vs. 37.56% in Class 1). No significant differences were found among the groups for education level or marital status (*p* > 0.05).

### 3.2. Network Structure and Centrality Analysis

We first constructed and visualized the psychological–safety network structures for the overall sample (a) and the three burnout latent profiles (b–d) to examine differences in node connection patterns (Figure 1). All networks included eight nodes. As shown in Figure 2, the EI analysis revealed distinct core drivers within each network. In the overall network, which comprised eight nodes and 20 nonzero edges, the network density was 71.4%. The skill factor (B4, EI = 1.134) emerged as the most central node, reflecting its pivotal role in linking psychological and safety‐related dimensions (Figure 2(a)).

FIGURE 1Network structure of psychological and safety factors across nurse burnout latent profiles. (a) Overall network, (b) Class 1: High Achievement Stable Group, (c) Class 2: High Efficiency Contradictory Group, and (d) Class 3: High Pressure Adaptive Group. Edge colors indicate the direction of association: Blue edges represent positive associations, and red edges represent negative associations. Edge thickness reflects the strength of the partial correlation between nodes.

**Figure:**
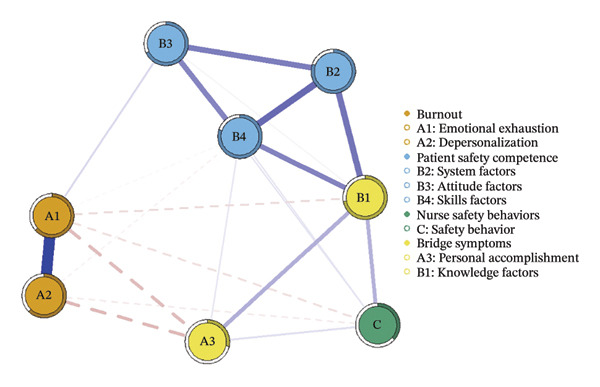
(a)

**Figure:**
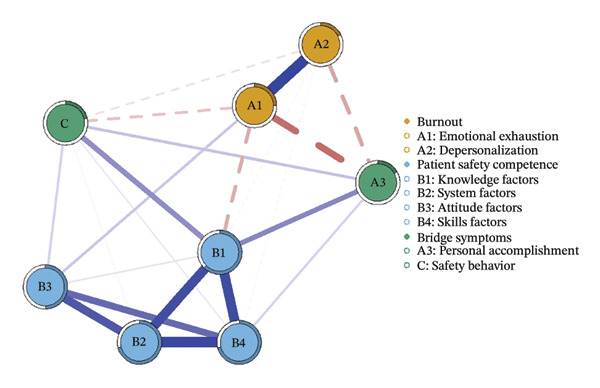
(b)

**Figure:**
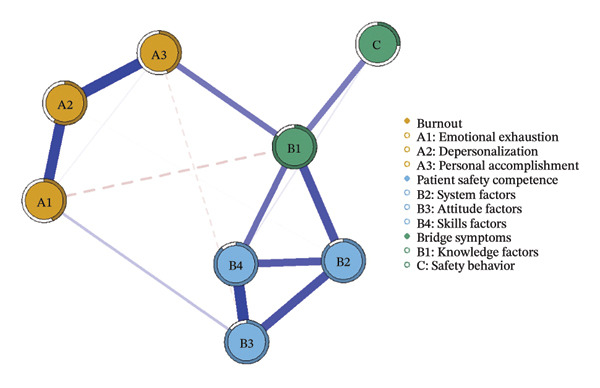
(c)

**Figure:**
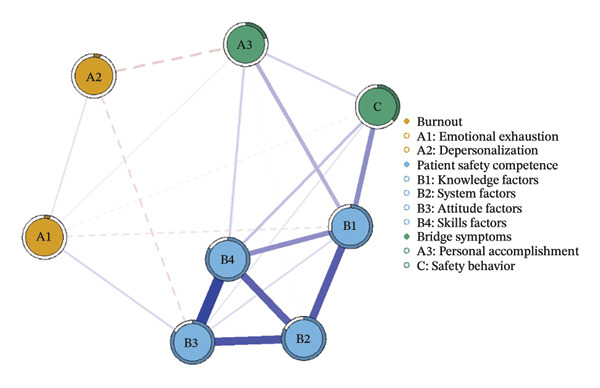
(d)

FIGURE 2Rankings of node centrality and bridge centrality. (a) Overall network, (b) Class 1: High Achievement Stable Group, (c) Class 2: High Efficiency Contradictory Group, and (d) Class 3: High Pressure Adaptive Group. A1: Emotional Exhaustion; A2: Depersonalization; A3: Personal Accomplishment; B1: Knowledge Factors; B2: System Factors; B3: Attitude Factors; B4: Skills Factors; C: Safety Behavior.

**Figure:**
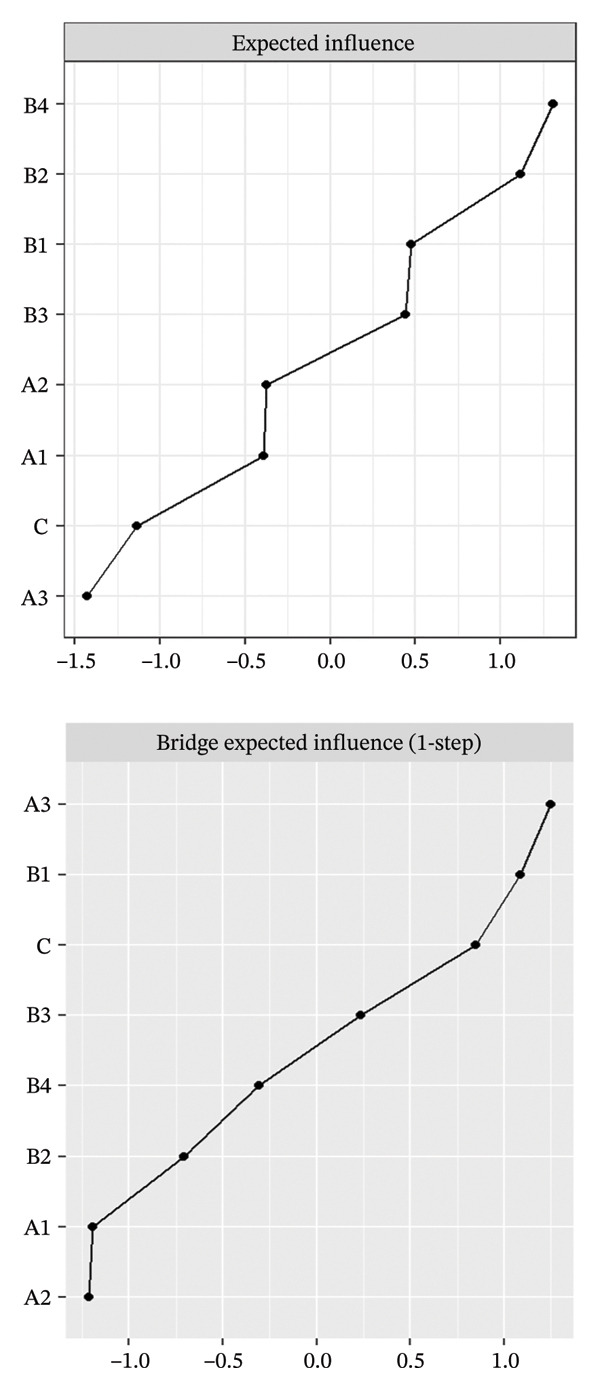
(a)

**Figure:**
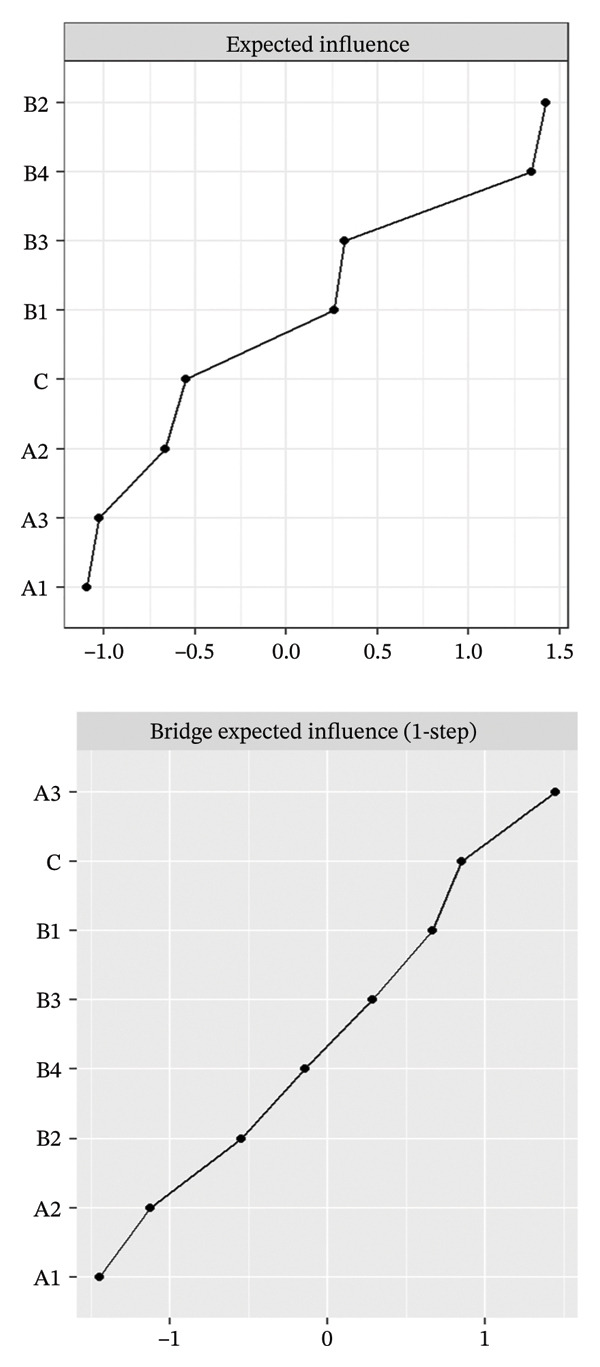
(b)

**Figure:**
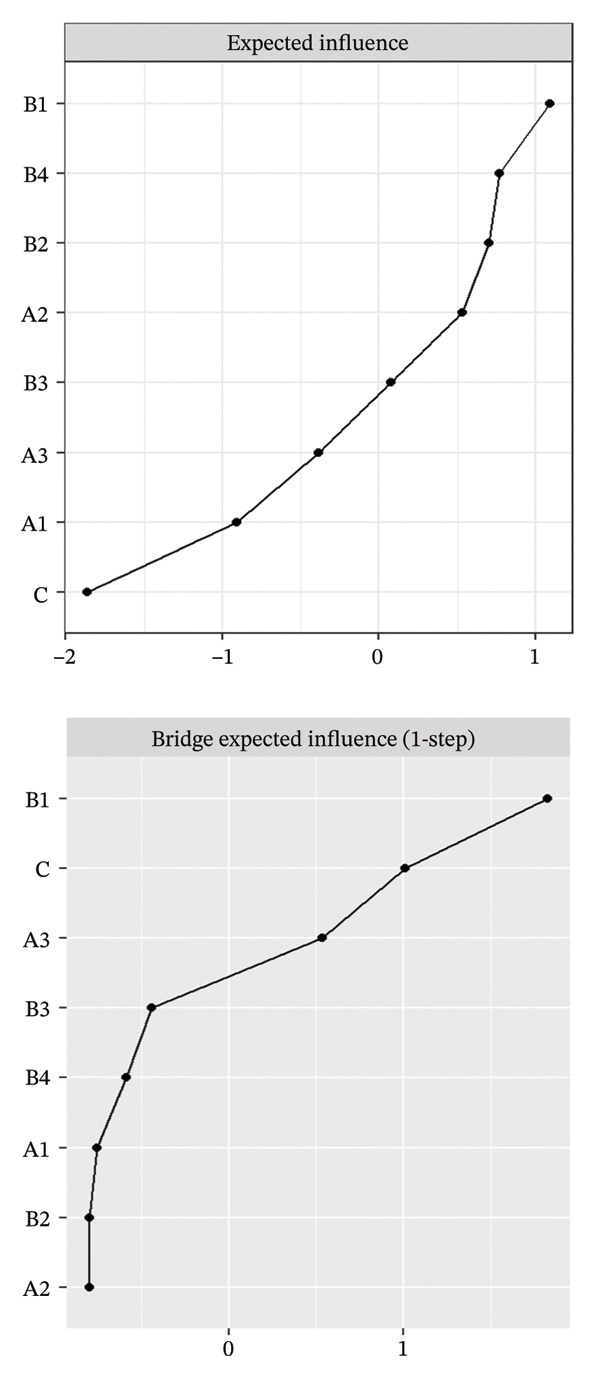
(c)

**Figure:**
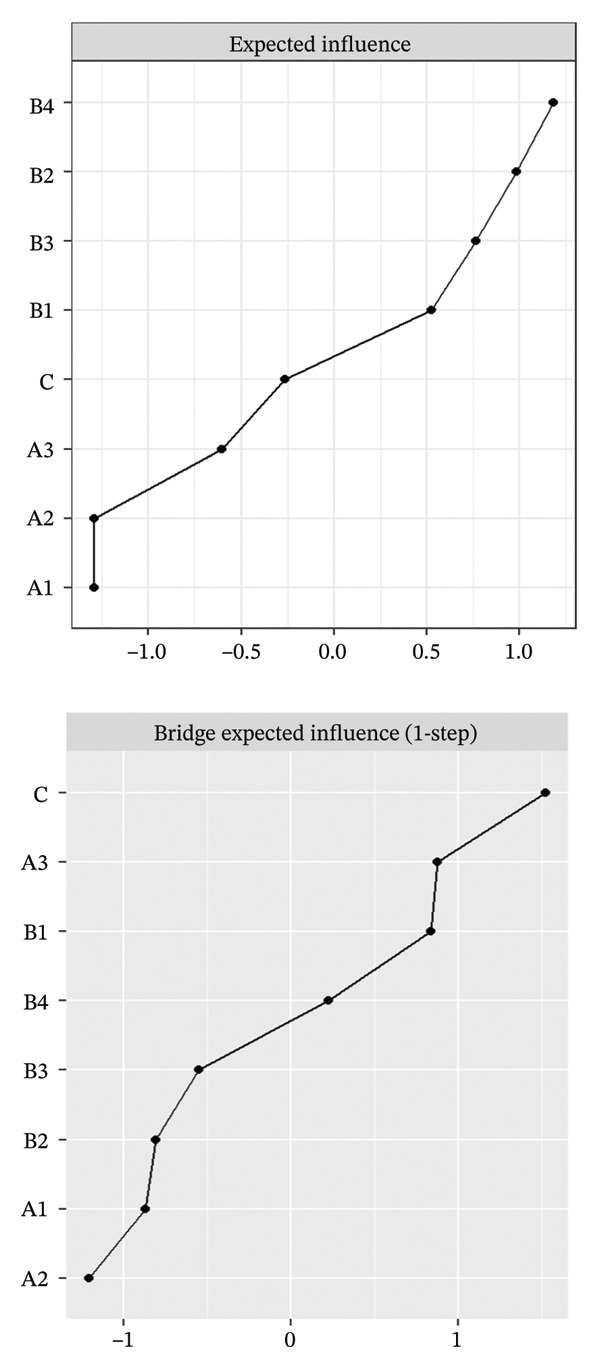
(d)

Among the subgroup networks, the “High Achievement Stable Group” (Class 1) demonstrated the highest network density (60.7%), with the system factor (B2, EI = 1.122) showing the strongest centrality. This finding highlights the critical influence of organizational systems in maintaining network stability within low‐risk groups (Figure 2(b)).

The “High Efficiency Contradictory Group” (Class 2) exhibited the sparsest network (density = 32.1%) but had the highest mean edge weight (0.118), suggesting that its connections were fewer but stronger. In this network, the knowledge factor (B1, EI = 1.168) served as the primary hub, indicating its dominant role and the cognitive core underlying high‐efficiency states (Figure 2(c)).

The “High‐Pressure Adaptive Group” (Class 3) showed a network density of 42.9%, consistent with the overall structure, with the skill factor (B4, EI = 1.139) remaining the most central node. This result underscores the continuing importance of skill competence in facilitating individual adaptation to high‐pressure environments (Figure 2(d)). Refer to Supporting Table S1 for the detailed EI values of all nodes across the overall network and burnout latent profiles.

### 3.3. Bridge Centrality Analysis

The bridge centrality analysis aimed to identify the key nodes that connect the burnout and safety communities. The lower panels of Figure 2 display the ranking of BEI for each node. In the overall psychological–safety network, personal accomplishment (A3, BEI = 0.319) and knowledge factors (B1, BEI = 0.292) showed the strongest cross‐community bridging effects, suggesting that these two nodes play crucial roles in linking and transmitting information between psychological and safety domains (Figure 2(a)).

Differences in the distribution of bridge nodes were observed across latent profiles. In the “High Achievement Stable Group” (Class 1), the main bridge nodes were personal accomplishment (A3, BEI = 0.321) and safety behavior (C, BEI = 0.229), indicating a strong interaction between positive achievement experiences and safety behavior (Figure 2(b)). In the “High Efficiency Contradictory Group” (Class 2), the bridge nodes were knowledge factors (B1, BEI = 0.394) and safety behavior (C, BEI = 0.275), suggesting that cognitive resources and behavioral responses serve as major mediating channels within the psychological–safety network (Figure 2(c)). In the “High Pressure Adaptive Group” (Class 3), the bridge nodes were safety behavior (C, BEI = 0.413) and personal accomplishment (A3, BEI = 0.230), showing that under high‐pressure conditions, safety behavior becomes the central hub connecting psychological and safety domains. In contrast, personal accomplishment continues to maintain a secondary bridging function (Figure 2(d)). Refer to Supporting Table S2 for detailed BEI values of all nodes across the overall network and burnout latent profiles.

**FIGURE 3:**
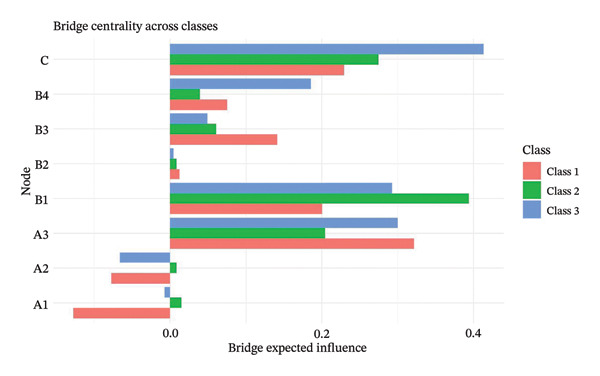
Comparison of bridge expected influence (BEI) among nodes across burnout latent profiles of nurses. Class 1: High Achievement Stable Group; Class 2: High Efficiency Contradictory Group; Class 3: High Pressure Adaptive Group. A1: Emotional Exhaustion; A2: Depersonalization; A3: Personal Accomplishment; B1: Knowledge Factors; B2: System Factors; B3: Attitude Factors; B4: Skills Factors; C: Safety Behavior.

The comparative results of bridge centrality (Figure 3) revealed that although the overall strength of bridge structures did not differ significantly between the high‐risk groups (Classes 2 and 3) and the healthy group (Class 1) according to the Wilcoxon paired test (*p* > 0.05), the functional roles of key nodes had shifted. The visualization clearly demonstrates that in the “High Efficiency Contradictory Group” (Class 2), the knowledge factor (B1) served as the most dominant bridging node connecting burnout and safety behavior, with the highest BEI value, surpassing the reliance on personal accomplishment (A3) observed in the healthy group. This finding suggests a mechanistic shift in the optimal intervention targets across different burnout profiles, highlighting the need for personalized interventions focusing on cognitive resources.

### 3.4. Network Stability and Accuracy Assessment

Network stability was evaluated using the CS coefficient, as shown in Figure 4. The CS test assesses whether the ranking of BEI and edge weights remains consistent when the sample size is reduced. For identifying intervention targets, the CS coefficients of BEI in the overall network and the three burnout latent classes ranged from 0.67 to 0.75, while the CS coefficients for edge weights were all 0.75. All CS values substantially exceeded the recommended threshold of 0.5, providing strong evidence that the centrality rankings are highly robust.

FIGURE 4Correlation of bridge expected influence and edge weights with the original sample. (a) Overall network; (b) Class 1: High Achievement Stable Group; (c) Class 2: High Efficiency Contradictory Group; (d) Class 3: High Pressure Adaptive Group.

**Figure:**
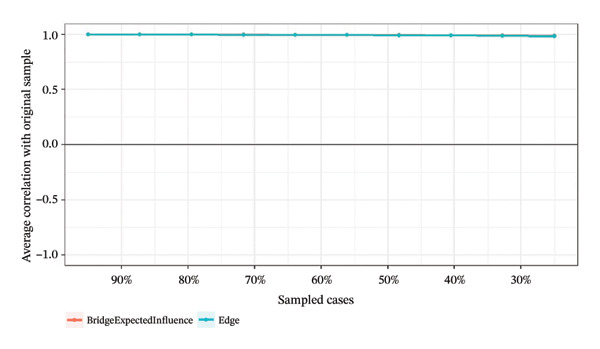
(a)

**Figure:**
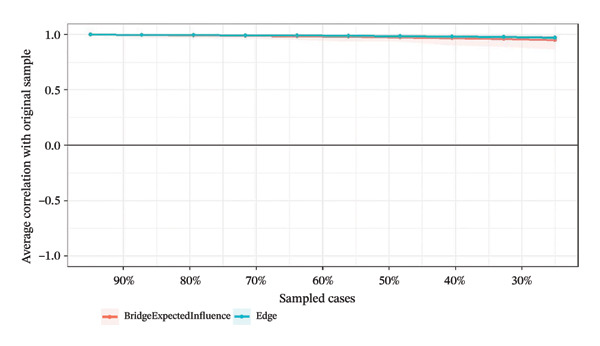
(b)

**Figure:**
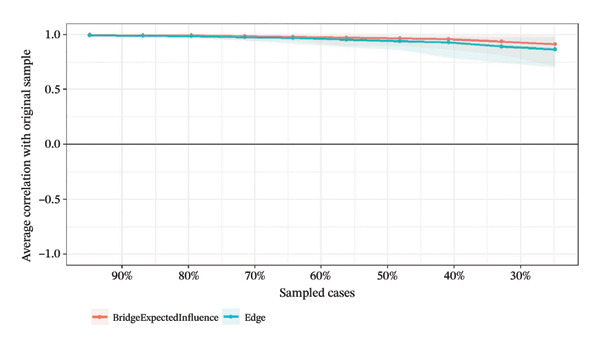
(c)

**Figure:**
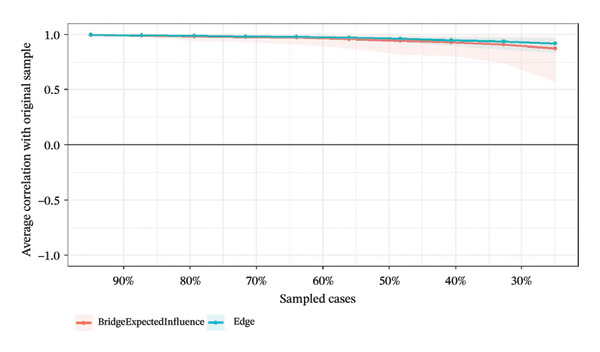
(d)

Network accuracy was assessed using a nonparametric bootstrap to calculate 95% CIs, as shown in Figure 5. The figure presents accuracy estimates for all network edge weights, with narrow gray intervals indicating high precision. The tight 95% CIs confirm that the edge weight estimates are precise and support the reliability of the significant differences reported in the NCT.

FIGURE 5Accuracy estimation of network edge weights and their 95% confidence intervals. (a) Overall network; (b) Class 1: High Achievement Stable Group; (c) Class 2: High Efficiency Contradictory Group; (d) Class 3: High Pressure Adaptive Group. A1: Emotional Exhaustion; A2: Depersonalization; A3: Personal Accomplishment; B1: Knowledge Factors; B2: System Factors; B3: Attitude Factors; B4: Skills Factors; C: Safety Behavior.

**Figure:**
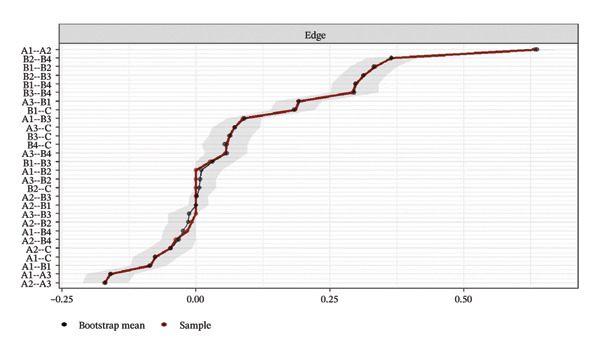
(a)

**Figure:**
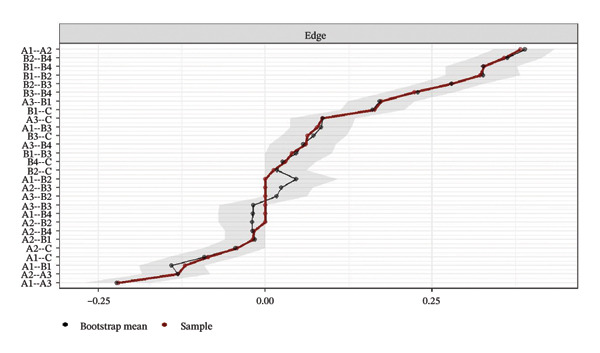
(b)

**Figure:**
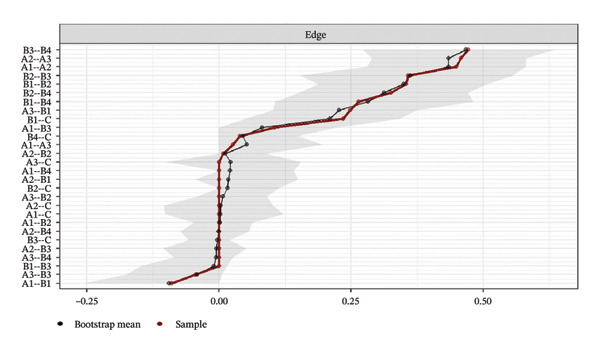
(c)

**Figure:**
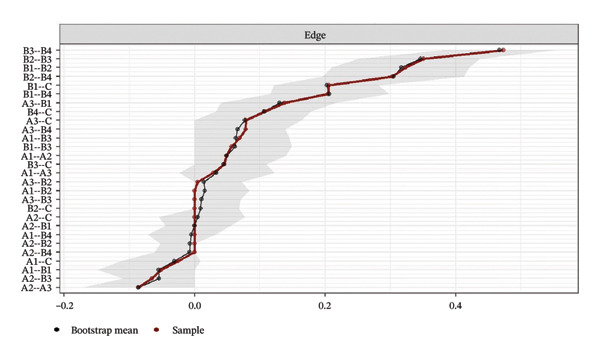
(d)

### 3.5. Network Comparison

The NCT results strongly indicate significant structural differences among various burnout latent profiles. When comparing the “High Efficiency Contradictory Group” (Class 2) with the “High Achievement Stable Group” (Class 1), the total network strength showed no notable difference (S, *p* = 0.781), but the network structure was notably different (M, *p* < 0.001), pointing to a mismatch rather than weaker mechanisms. Key edge differences were found in depersonalization and personal accomplishment (A2–A3, *p* < 0.001) and emotional exhaustion and system factors (A1–B2, *p* = 0.043), highlighting a distinct impact of systemic stress on emotional responses. Additionally, the link between attitude factors and skills factors (B3–B4) also varied significantly (*p* = 0.033).

By contrast, the “High Pressure Adaptive Group” (Class 3) showed even more pronounced structural differences compared with the “High Achievement Stable Group” (Class 1). The two networks differed significantly in overall structure (M, *p* < 0.001), and the key edge differences in Class 3 highlighted both impairments in safety mechanisms and the absence of protective connections. Specifically, emotional exhaustion and knowledge factors (A1–B1, *p* = 0.004) indicated the loss of protective cognitive links; system factors and skills factors (B2–B4, *p* = 0.002) and attitude factors and skills factors (B3–B4, *p* < 0.001) reflected systematic defects within the safety competence subnetwork; and emotional exhaustion and safety behavior (A1–C, *p* = 0.047) suggested a unique direct negative impact of emotional exhaustion on safety behavior in this group. These results (Supporting Table S3) provide strong evidence that high‐risk profiles exhibit reorganized and functionally weakened network structures, offering robust statistical support for the development of personalized interventions.

## 4. Discussion

This study integrated LPA and network analysis to map the complex interplay between burnout, safety competence, and safety behavior. Our results validate H1, H2, and H3, confirming that these constructs form multidimensional networks that vary across burnout profiles. From the SCT perspective, safety competence emerged as both a central hub for professional performance (H1) and a critical bridge mediating the burnout‐behavior link (H2). The observed heterogeneity in network stability across the three classes underscores the necessity for profile‐specific management strategies (H3), moving beyond “one‐size‐fits‐all” approaches to ensure nursing safety.

### 4.1. Network Features and Centrality

The overall psychological–safety network showed high connectivity, indicating close interdependence between nurses’ internal states, competencies, and behaviors. From the perspective of SCT’s triadic reciprocal determinism, the “central nodes” identified in our network analysis can be interpreted as the dominant drivers within the personal or environmental domains that exert the strongest influence on the behavioral system [16]. In this study, the skills dimension (B4) emerged as the most central node. This highlights that clinical capabilities function as critical personal factors that not only underpin nursing quality but also facilitate self‐regulation and adaptive safety behaviors when navigating environmental stressors [30].

This study identified three burnout subtypes through LPA, aligning with the growing emphasis on burnout heterogeneity in nursing research [31, 32]. Profile‐specific analysis revealed distinct patterns that resonate with SCT. In the “High Achievement Stable Group” (Class 1), the network exhibited the highest stability with system support (B2) as the core hub; this illustrates how a supportive environment (Environmental Factor) facilitates the internalization of safety behaviors, reinforcing SCT’s environment–behavior interaction [33]. The “High Efficiency Contradictory Group” (Class 2) was characterized by a reliance on cognitive resources to sustain safety behavior under high workloads. This pattern exemplifies SCT’s triadic interaction, where personal cognitive factors drive behavioral outcomes to meet environmental demands, even when personal regulation is strained [34]. Conversely, in the “High Pressure Adaptive Group” (Class 3), weakened network connectivity and diminished core nodes indicate depleted personal resources and compromised environmental support. This is consistent with SCT’s premise that behavioral performance suffers when the reciprocal triadic interaction is disrupted.

### 4.2. Bridge Centrality Network Comparison

Bridge analysis further elucidates the mechanisms within SCT by identifying the pathways through which burnout affects safety performance. In the overall network and the “High Achievement Stable Group” (Class 1), personal accomplishment (A3) exhibited the highest BEI, effectively linking internal psychological states to safety behaviors. This aligns with SCT, as self‐efficacy and the perceived meaningfulness of work act as personal factors that modulate motivation and behavioral enactment [35, 36].

In the “High Efficiency Contradictory Group” (Class 2), knowledge (B1) served as both a core hub and the strongest bridge. This emphasizes that cognitive personal factors are the primary mediators for translating safety concepts into observable behaviors, particularly under high‐demand conditions where emotional regulation may be strained. Conversely, the “High Pressure Adaptive Group” (Class 3) showed a relative enhancement of safety behavior nodes as bridges. This suggests that when personal resources are depleted, environmental cues and strict adherence to protocols become the primary drivers of behavior, reflecting a shift toward environmental–behavioral dependency. Notably, the smaller sample size of Class 2 necessitates a cautious interpretation of these findings, which should be considered exploratory.

### 4.3. Clinical Implications

To bridge the gap between theory and practice, we propose a stratified intervention framework that targets the specific network hubs of each burnout profile. For the “High Achievement Stable Group” (Class 1), managers should leverage their high personal accomplishment by appointing them as “Safety Preceptors” or involving them in developing departmental protocols. This “leadership empowerment” model reinforces their internal motivation and utilizes their stability to anchor the ward’s safety culture [37].

For profiles characterized by higher strain, interventions must be more precision‐guided. For the “High Efficiency Contradictory Group” (Class 2), the priority is “cognitive load reduction” through mobile‐based decision support systems and VR‐based crisis simulations, which help automate safety responses and prevent cognitive exhaustion [38]. Conversely, for the “High Pressure Adaptive Group” (Class 3), focus should shift to “psychological scaffolding” to compensate for resource depletion. This involves implementing restorative mindfulness‐based stress reduction programs to help nurses rebuild internal psychological resilience [39]. To supplement this, managers should optimize clinical workflows by integrating standardized, simplified safety checklists and automated reminders. These environmental “scaffolds” ensure that safety compliance is maintained through system‐level support rather than relying solely on the nurse’s exhausted internal regulation.

Finally, nursing administrations should operationalize bridge nodes (knowledge and personal accomplishment) as dynamic screening indicators. Incorporating these metrics into annual audits allows for an “early‐warning system”: A significant decline in these specific scores can trigger preemptive support, such as career counseling or temporary workload adjustments. This proactive approach enables managers to intervene before a nurse transition from a stable state to a high‐burnout profile, ensuring long‐term professional sustainability.

### 4.4. Limitations

Several limitations should be acknowledged. First, the cross‐sectional design precludes causal inference between burnout and safety behavior; longitudinal studies are needed to capture dynamic relationships. Second, reliance on self‐reported measures may introduce common method bias, and future research should incorporate objective behavioral or performance indicators. Third, convenience sampling may limit the generalizability of the findings beyond oncology nurses in tertiary hospitals.

Additionally, sample sizes differed across latent burnout profiles, particularly the relatively small High Efficiency Contradictory Group (Class 2). Although this reflects naturally occurring profile distributions, findings for this subgroup should be interpreted with caution and considered exploratory, warranting replication in larger or more balanced samples. Finally, further validation is needed to confirm the stability of network structures and the interpretability of bridge metrics.

## 5. Conclusion

By integrating LPA and network analysis, this study systematically revealed the reconstruction characteristics of the psychological–safety network in oncology nurses across different burnout types. The findings indicate that safety competence occupies a central position in all profile networks, serving as a key hub for maintaining psychological balance and promoting safe behavior. Personal accomplishment and knowledge factors consistently bridge burnout and safety behavior across profiles. Different burnout types exhibited distinct network patterns: The “High Efficiency Contradictory Group” displayed a “high cognition–high consumption” pattern, while the “High Pressure Adaptive Group” exhibited a “resource depletion–weakened connections” pattern. These results deepen our understanding of nurse burnout mechanisms and provide theoretical and practical evidence for targeted and precise interventions, guiding nursing managers to enhance nurse safety behaviors and support professional sustainability in high‐pressure environments.

## Author Contributions

Fengyan Ma conceived the study, developed the methodology, performed the formal analysis, prepared the original draft, and managed the project. Zhao Luo performed the formal analysis and prepared the original draft. Yajing Zhu conceptualized the study, contributed to the original draft preparation, and conducted the survey. Lu Liu contributed to the conceptualization and performed the survey. Helin Chen performed the investigation and organized the data. Yan Liu acquired the resources, reviewed and revised the manuscript, and provided supervision. Fan Zhang acquired the funding, provided resources, and performed the review and supervision of the manuscript. Fengyan Ma, Zhao Luo, and Yajing Zhu are the co‐first authors.

## Funding

Financial support for this study was provided by noncommercial academic sources, including the CAMS Innovation Fund for Medical Sciences (Grant No. 2024‐I2M‐C&T‐B‐053) and the National High Level Hospital Clinical Research Funding (No. 80102022501).

## Conflicts of Interest

The authors declare no conflicts of interest.

## Supporting Information

The following supporting information is available online:

Table S1: Network Expected Influence Metrics.

Table S2: Network Bridge Expected Influence Metrics.

Table S3: Summary of Edge Differences Across Burnout Latent Profiles.

## Supporting information


**Supporting Information** Additional supporting information can be found online in the Supporting Information section.

## Data Availability

The datasets used during this study are available from the corresponding author upon reasonable request.
