# Supplementary material for: Network Analysis of Burnout and Safety Competence Among Oncology Nurses: A Secondary Study to Identify Bridge Targets for Precision Interventions
Source: J Nurs Manag. 2026 Mar 23;2026:5604987. doi: 10.1155/jonm/5604987 (PMC13140357; doi:10.1155/jonm/5604987)
Supplement: Supplementary file 1 — Supporting Information Additional supporting information can be found online in the Supporting Information section. [file JONM-2026-5604987-s001.docx]

**Supplementary Material**

Table S1: Network Expected Influence Metrics

Table S2: Network Bridge Expected Influence Metrics

Table S3: Summary of Edge Differences Across Burnout Latent Profiles

| **Table S1: Network Expected Influence Metrics** | | | | |
| --- | --- | --- | --- | --- |
| **Variable** | **Overall Network** | **High Achievement Stable Group** | **High Efficiency Contradictory Group** | **High Pressure Adaptive Group** |
| A1 | 0.551 | 0.178 | 0.546 | 0.000 |
| A2 | 0.557 | 0.339 | 0.994 | 0.000 |
| A3 | 0.195 | 0.203 | 0.708 | 0.317 |
| B1 | 0.849 | 0.688 | **1.168** | 0.837 |
| B2 | 1.068 | **1.122** | 1.047 | 1.048 |
| B3 | 0.837 | 0.708 | 0.853 | 0.947 |
| B4 | **1.134** | 1.093 | 1.067 | **1.139** |
| C | 0.296 | 0.382 | 0.249 | 0.475 |

Note, A1: Emotional Exhaustion; A2: Depersonalization; A3: Personal Accomplishment; B1: Knowledge Factors; B2: System Factors; B3: Attitude Factors; B4: Skills Factors; C: Safety Behavior.

| **Table S2: Network Bridge Expected Influence Metrics** | | | | |
| --- | --- | --- | --- | --- |
| **Variable** | **Overall Network** | **High Achievement Stable Group** | **High Efficiency Contradictory Group** | **High Pressure Adaptive Group** |
| A1 | -0.089 | -0.127 | 0.015 | -0.007 |
| A2 | -0.093 | -0.077 | 0.008 | -0.066 |
| A3 | **0.319** | **0.321** | 0.204 | 0.300 |
| B1 | 0.292 | 0.200 | **0.394** | 0.293 |
| B2 | -0.008 | 0.013 | 0.008 | 0.004 |
| B3 | 0.150 | 0.142 | 0.061 | 0.049 |
| B4 | 0.059 | 0.075 | 0.040 | 0.185 |
| C | 0.253 | 0.229 | 0.275 | **0.413** |

Note, A1: Emotional Exhaustion; A2: Depersonalization; A3: Personal Accomplishment; B1: Knowledge Factors; B2: System Factors; B3: Attitude Factors; B4: Skills Factors; C: Safety Behavior.

| **Table S3: Summary of Edge Differences Across Burnout Latent Profiles** | | | | |
| --- | --- | --- | --- | --- |
| **Edge (Var1−Var2)** | **Class 2 vs. Class 1** | | **Class 3 vs. Class 1** | |
|  | ***P*** | **E Statistic** | ***P*** | **E Statistic** |
| A1 - A2 | **0.026** | 0.209 | **<0.001** | 0.292 |
| A1 - A3 | 0.499 | 0.060 | 0.082 | 0.099 |
| A2 - A3 | **<0.001** | 0.502 | 0.797 | 0.012 |
| A1 - B1 | 0.413 | 0.087 | **0.004** | 0.118 |
| A2 - B1 | 0.959 | 0.022 | 1.000 | 0.000 |
| A3 - B1 | 0.083 | 0.143 | 0.806 | 0.012 |
| A1 - B2 | **0.043** | 0.070 | 0.408 | 0.049 |
| A2 - B2 | 0.793 | 0.026 | 0.666 | 0.020 |
| A3 - B2 | 0.325 | 0.033 | 0.287 | 0.033 |
| B1 - B2 | 0.603 | 0.053 | 0.469 | 0.048 |
| A1 - B3 | 0.864 | 0.019 | 0.288 | 0.045 |
| A2 - B3 | 0.143 | 0.015 | **0.016** | 0.078 |
| A3 - B3 | 0.915 | 0.023 | 0.138 | 0.060 |
| B1 - B3 | 0.427 | 0.024 | 0.839 | 0.011 |
| B2 - B3 | 0.381 | 0.082 | 0.222 | 0.074 |
| A1 - B4 | 0.070 | 0.061 | 0.168 | 0.046 |
| A2 - B4 | 0.692 | 0.020 | 0.621 | 0.020 |
| A3 - B4 | 0.215 | 0.078 | 0.912 | 0.005 |
| B1 - B4 | 0.970 | 0.003 | 0.158 | 0.082 |
| B2 - B4 | 0.109 | 0.144 | **0.002** | 0.188 |
| B3 - B4 | **0.033** | 0.221 | **<0.001** | 0.261 |
| A1 - C | 0.286 | 0.065 | **0.047** | 0.067 |
| A2 - C | 0.577 | 0.067 | 0.374 | 0.050 |
| A3 - C | 0.151 | 0.096 | 0.561 | 0.030 |
| B1 - C | 0.262 | 0.092 | 0.969 | 0.002 |
| B2 - C | 0.722 | 0.019 | 0.254 | 0.057 |
| B3 - C | 0.153 | 0.059 | 0.981 | 0.001 |
| B4 - C | 0.257 | 0.071 | 0.935 | 0.005 |

Note, Class 1: High Achievement Stable Group; Class 2: High Efficiency Contradictory Group; Class 3: High Pressure Adaptive Group.
A1: Emotional Exhaustion; A2: Depersonalization; A3: Personal Accomplishment; B1: Knowledge Factors; B2: System Factors; B3: Attitude Factors; B4: Skills Factors; C: Safety Behavior.
